# Therapeutic efficacy of arthroscopy-assisted transosseous fixation with the Versalok suture anchor for tibial eminence fractures in adults

**DOI:** 10.1097/MD.0000000000026284

**Published:** 2021-06-11

**Authors:** Yuanshi She, Dongsheng Guo, Guangxiang Chen, Youjia Xu

**Affiliations:** a Department of Orthopedics, Second Affiliated Hospital of Soochow University, No.1055 Sanxiang Road, Suzhou; b Department of Orthopedics, The First People's Hospital of Lianyungang, Lianyungang; c Department of Orthopedics, Nanjing Medical University Affiliated Suzhou Hospital (Suzhou Municipal Hospital), Suzhou, Jiangsu, China.

**Keywords:** arthroscopy, fracture fixation, fractures, tibia

## Abstract

To investigate the clinical outcomes of arthroscopy-assisted transosseous fixation of tibial eminence fractures with the Versalok suture anchor in adults.

A total of 23 adult cases of tibial eminence fractures treated between June 2016 and March 2019 were retrospectively analyzed. The results of the preoperative drawer test and Lachman test were positive. Radiography and computed tomography were performed before and after the procedure. Magnetic resonance imaging was performed in every patient after admission. Arthroscopy-assisted fracture reduction and Orthocord high-strength suture fixation with two Versalok anchors were performed in all the patients. The International Knee Documentation Committee scale and the Lysholm Knee Scoring Scale were used to evaluate outcomes during the follow-up period. Additionally, the KT-2000 knee stability test was performed.

At the final follow-up, all the fractures had proceeded to bony union and no wound infection was observed. The average Lysholm Knee Score of the affected knees was 93.1 (range, 90–98), which was not significantly different from that of the healthy knees (*t* = 0.732, *P* = .132). Based on the International Knee Documentation Committee scale results, 21 patients were graded as normal and the other 2 patients were graded as nearly normal. The KT-2000 test showed that the anterior displacement of the affected side and the healthy side was less than 3.6 mm in all cases.

The outcomes indicated firm fixation and good fracture healing with minimal trauma. Thus, arthroscopy-assisted transosseous fixation with Versalok suture anchors for adult tibial eminence fractures seems to have satisfactory clinical outcomes.

## 1 Introduction

Tibial eminence fractures typically occur at the bony insertion of the anterior cruciate ligament (ACL), and they lead to pain, limited extension, and anterior instability of the knee. Axial rotation is the major pathological cause of tibial eminence fractures, and the mechanism is similar to that of ACL rupture in adults. Additionally, tibial eminence fractures may also be caused by direct injury or joint hyperextension.^[1]^ The most common activity associated with tibial eminence fractures is falling off a bicycle. Very recently, skiing and motor vehicle accidents have become increasingly common risk factors.^[2,3]^

Arthroscopic reduction and fixation techniques have been described by McLennan^[4]^ as early as 1982 for the treatment of type II–IV fractures based on the modified Meyers-McKeever classification, and these techniques have demonstrated biomechanical and clinical effectiveness.^[5–7]^ Some of the advantages of these techniques are minimal invasiveness, effective pain control, early rehabilitation, and simultaneous treatment of concomitant intra-articular injury (i.e., meniscus tear and chondral lesion).^[1]^ According to a literature review, diverse methods have been reported with the use of screws, wires, and suture anchors.^[8–10]^ However, there is no consensus on the most optimal tool for fracture treatment. For example, cancellous screws cannot be used for comminuted fractures or small fragments,^[11]^ and have a significantly lower median peak failure load in load-to-failure testing.^[12]^ Additionally, complications such as limited range of motion and anterior instability of the knee have been described in several reports.^[9,13]^

The present study aims to retrospectively investigate the clinical outcomes of arthroscopy-assisted transosseous fixation with Versalok suture anchors for the correction of avulsion fracture of the ACL tibial insertion and the importance of preoperative magnetic resonance imaging (MRI).

## 2 Materials and methods

### 2.1 Participants

This retrospective study was approved by the Health Sciences Institutional Review Board of our hospital, and written consent was obtained from all the participants.

**Figure 1:**
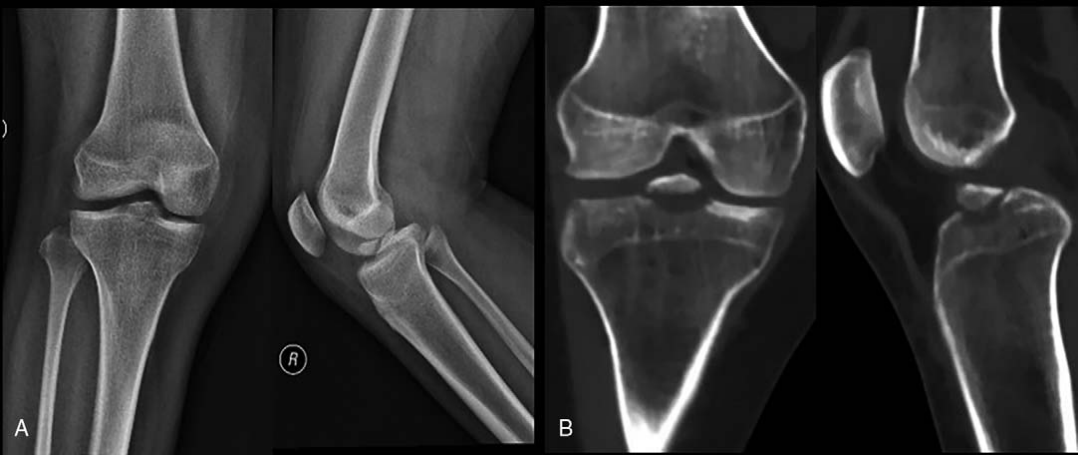
Preoperative radiograph (A) and CT (B) findings for a type III typical tibial eminence fracture according to modified Meyers-McKeever classification.

Patients with type II, III, and IV fractures, based on the modified Meyers-McKeever classification (Fig. 1), and positive results for Lachman test and the anterior draw test under gentle maneuvering in the emergency clinic were found to be eligible and were recruited in this trial. MRI was preoperatively performed to evaluate meniscal tear, and ACL and other soft-tissue injuries. Patients with tibial plateau fractures and concomitant midsubstance injury of the ACL were excluded.

### 2.2 Surgical technique

**Figure 2:**
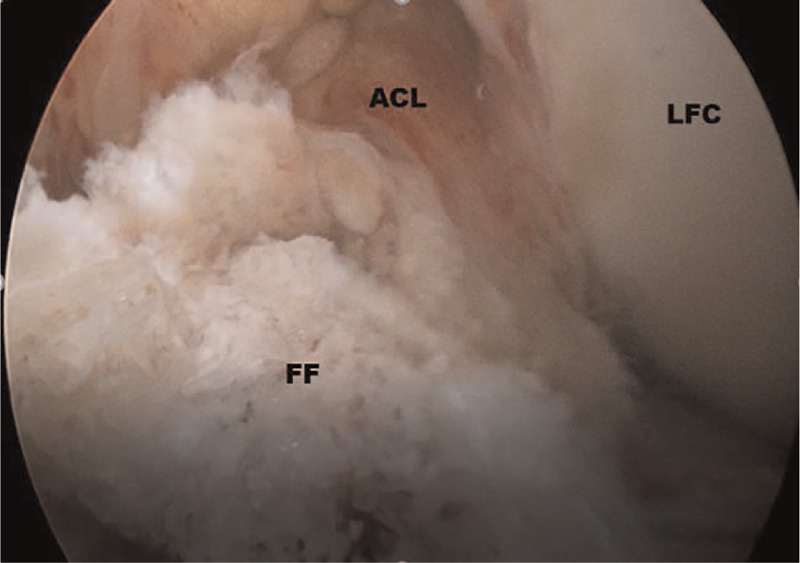
Debriding of the hematoma of the fracture bed with an arthroscopic shaver. (ACL = anterior cruciate ligament, FF = fracture fragment; LFC = lateral femoral condyle).

With the patient in a supine position and the lower limb on the operating table, the knee was fully extended and flexed to 120 degrees. An air tourniquet was attached on the upper side of the affected thigh. The high anteromedial and anterolateral approach (parallel to the inferior surface of the patella) was adopted. Diagnostic arthroscopy was performed to re-confirm the diagnosis and identify any concomitant pathologies, such as meniscal tear, loose body, midsubstance ACL tear, or femoral avulsion fracture of the ACL. The need for meniscus repair was determined according to tear location and tear type. Soft tissues preventing observation and reduction were disengaged, and the continuity of the intermeniscal ligament was maintained to ensure the stability of the anterior horns of the menisci. Then, the hematoma of the fracture bed was debrided with an arthroscopic shaver (Fig. 2). It was necessary to remove sclerotic bone from both the fracture bed and the undersurface of the avulsed fragment for ununited fractures with an arthroscopic burr. The fracture bed needed to be properly deepened to facilitate bone reduction, and a trial reduction with a probe was necessary before fixation.

A 2-cm longitudinal incision was made medial and inferior to the tibial tubercle down to the bone. A 1.5-mm K-wire was inserted into the joint with the outlets located anteromedial and anterolateral to the tibial fragment, with the help of an ACL tibial guide. The distance between the entry points was 1.5 cm. Then, two bone tunnels were drilled with a 4.5-mm drill and a guide wire.

**Figure 3:**
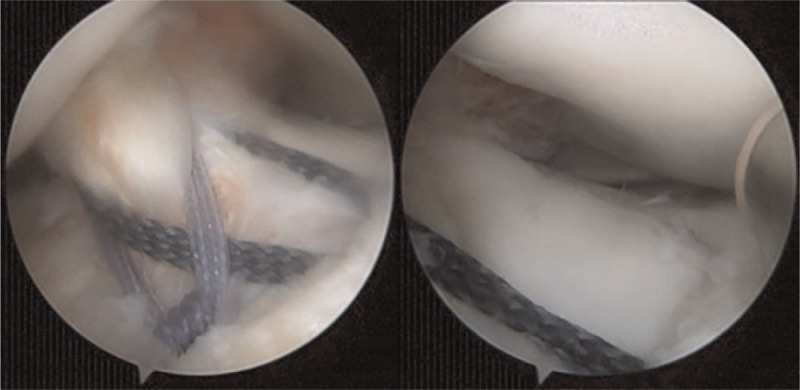
Satisfactory fracture reduction and fixation in the anteroposterior and lateral planes with Versalok suture anchors.

A suture lasso was introduced through the high-anteromedial or high-anterolateral portal and toward the posterior one-third of the ACL tibial insertion. Next, four-strand Orthocord high-strength sutures were advanced through the suture passer and made to penetrate the insertion of the ACL with two strands per side. Every two-strand suture was knotted and crossed around the ACL adjacent to the fracture fragment and was retrieved through the bone tunnel. An arthroscope was introduced into the joint to confirm anatomic reduction of the fracture while tensioning the sutures with the Versalok gun (Fig. 3). With the knee in extension and the application of a posterior drawer force, the sutures were locked into the bone near the entry of the tunnels. Finally, the incisions were sutured, and the knee was placed in a hinged knee brace and locked in extension.

### 2.3 Postoperative protocol and follow-up

A hinged brace was used to maintain full extension of the knee for the first 3 weeks after the procedure, and weight-bearing was forbidden. In the next 4 to 6 weeks, a 0 to 90° range of motion and touchdown weight bearing with crutches were allowed for the affected knee. Full weight-bearing and full range of motion were allowed at approximately 6 weeks after the procedure.

### 2.4 Clinical evaluation

**Figure 4:**
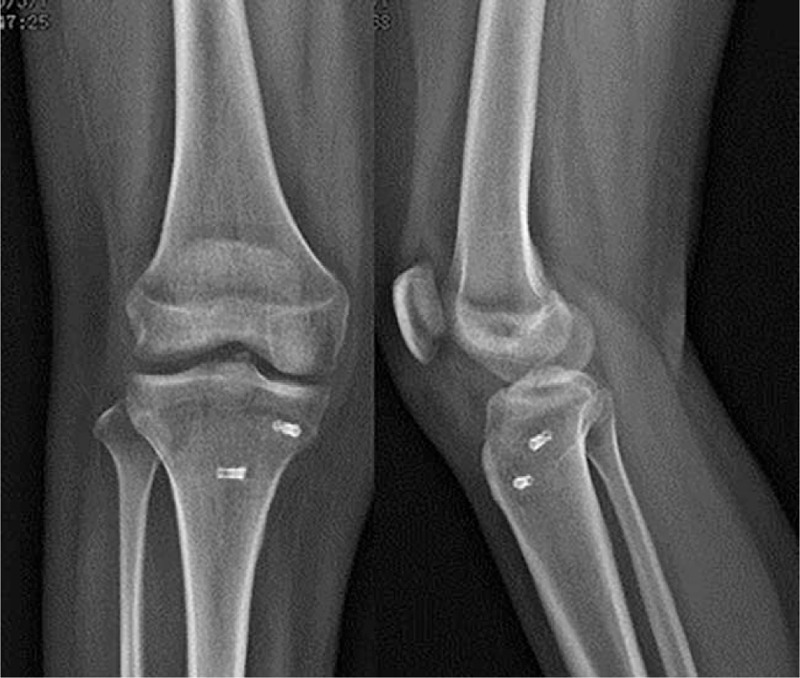
Radiograph showing satisfactory healing of the fracture.

Patient demographic data, including age, sex, time to surgical intervention, and other concomitant injuries, were collected from the admission records. Radiography, computed tomography, and MRI were performed before and after the procedure. Radiographs were obtained for examining fracture type and fracture recovery (Fig. 4). At the last follow-up, the International Knee Documentation Committee (IKDC) knee ligament examination form, which included objective evaluations and subjective scores, and the Lysholm Knee Scoring Scale were filled in and documented. The KT-2000 test was performed to assess the stability of the knee.

### 2.5 Statistical analysis

All statistical analyses were performed with the SPSS software (Version 17.0; SPSS, Chicago, IL). The data are presented as mean ± standard deviation. The statistical significance of differences in continuous data was compared using a paired *t*-test. All statistical data were two-sided and were evaluated at a 5% level of significance.

## 3 Results

**Figure 5:**
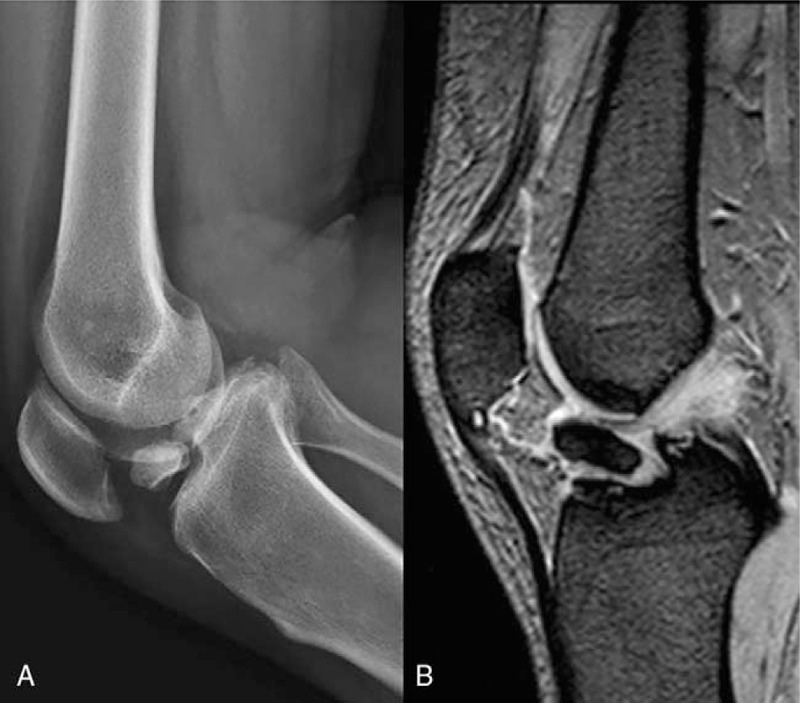
Preoperative radiograph and MRI findings in a case of ununited tibial eminence fracture. MRI = magnetic resonance imaging.

**Figure 6:**
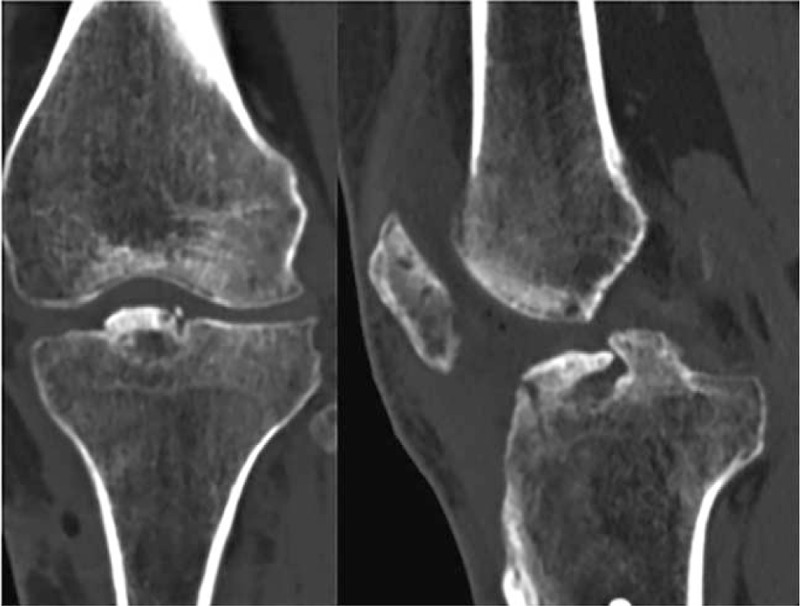
Postoperative CT scan showing satisfactory fracture reduction and healing in the case of ununited tibial eminence fracture. CT =computed tomography.

A total of 23 eligible cases with ACL tibial avulsion treated between June 2016 and March 2019 were retrospectively analyzed. The average age of the cohort was 38.3 years. The mean time from injury to surgical intervention was 6.5 days. Radiographs showed that the fractures were well reduced in all cases (Table 1), and all fractures had proceeded to bony union at the follow-up examination. In the present cohort, there was one case of ununited tibial eminence fracture (Fig. 5) in which good fracture union was achieved after surgery (Fig. 6). No wound infection was observed in any of the cases.

**Table 1:** Patient demographic data.

Demographic variables	Mean	Range
Follow-up time (mo)	8.5	6–14
Age (yr)	38.3	22–63
Interval between injury and procedure (d)	6.5	2–123
Gender (n)		
Male	8	
Female	15	
Fracture classification (n)		
Type II	3	
Type III	16	
Type IV	4	

At the final follow-up, the average Lysholm score of the affected knee was 93.1 (range, 90–98), which was not significantly different from that of the healthy side (*t *= 0.732, *P* = .132). With regard to the IKDC objective evaluation, 21 patients were graded as normal and 2 were graded as nearly normal, while the mean IKDC subjective score of the affected knee was 93.7 (range, 88–97). The KT-2000 test showed that anterior displacement of the affected side and the healthy side was less than 3.6 mm (standard deviation  = 0.8) in all cases (Table 2).

**Table 2:** Patient outcomes.

	Mean	Range
Lysholm score	93.1	90–98
IKDC objective evaluation		
Normal (n)	21	
Nearly normal (n)	2	
IKDC subjective score	93.7	88–97
Side-to-side distance on the KT-2000 test (mm)	2.8	2.0–3.6

IKDC = International Knee Documentation Committee.

## 4 Discussion

The findings of this retrospective study show that arthroscopy-assisted transosseous fixation with the Versalok suture anchor was minimally invasive and resulted in firm fracture union and good knee functional recovery in adults with type II to IV tibial eminence fractures.

In the present study, we achieved satisfactory clinical outcomes with arthroscopy-assisted reduction and fixation using Versalok anchors. The average Lysholm score of the affected knee was 93.1, which was not significantly different from that of the healthy knee. Furthermore, 21 patients were graded as normal and 2 as nearly normal at the final follow-up with the IKDC scale. It has previously been reported that arthroscopic suture fixation of tibial eminence fractures is a promising technique that leads to ideal outcomes.^[1]^ Additionally, several studies have found that suture fixation is stronger than screw fixation,^[12,14–16]^ and can be used to effectively fix small or comminuted fragments.^[17]^ According to all these findings, we recommend the use of arthroscopy-assisted transosseous fixation with the Versalok suture anchor for tibial eminence fracture in adults.

In our procedure, we used the Versalok gun to tension four strands of high-strength Orthocord sutures; in this way, uniform force was applied on the fragment and the stability of fixation was improved. Accordingly, the KT-2000 test showed that the laxity grade was less than 3.6 mm in all cases. A previous study^[9]^ pointed out that up to 44% of patients exhibit knee instability on the pivot-shift test, and 13% present with increased laxity with a KT-1000 side-to-side difference greater than 5 mm. Similarly, Kocher et al^[18]^ reported that the result of the Lachman test indicated abnormality in five out of six patients, with two exhibiting a pivot shift and four exhibiting an increase in the KT-1000 difference. Although persistent laxity rarely impairs functional outcomes, the low incidence of anterior instability in our report is a good outcome and might be attributed to the anatomic reduction of the fracture and rigid fixation.

Preoperative MRI is essential for appropriate diagnosis of avulsion fractures of the ACL tibial insertion. Based on the MRI findings, in this study, several patients with concomitant midsubstance ACL tears who had undergone sequential ACL reconstructions or augmentations were excluded. This is in agreement with the inclusion criteria of similar reports.^[19–21]^ Additionally, simultaneous femoral origin avulsion of the ACL has also been reported by Uhorchak et al.,^[22]^ and Noyes et al.^[23]^ noted that the incidence rate of combined ligamentous failure and tibial eminence fracture is up to 14%. In such cases, simple fixation of the fracture may further aggravate the ACL injury and increase the instability of the knee. Therefore, preoperative MRI is necessary for effectively identifying cases that require ACL reconstructions or augmentations.

Ununited tibial eminence fracture is less common and is rarely reported, but several treatment methods have been established previously.^[24–26]^ In our trial, one case of ununited tibial eminence fracture was fixed with the Versalok suture anchor arthroscopically, and good fracture union and normal knee function were achieved. During surgery in such cases, several key points need to be considered, as follows.

(1) The anterior intermeniscal ligament in the fracture needs to be pulled out or partially debrided for fracture reduction.^[24]^
(2) Placing a traction suture around the intermeniscal ligament is recommended to allow adequate visualization and to facilitate reduction of the avulsed bone fragment into its bed when necessary.^[27]^ However, maintaining the continuity of the intermeniscal ligament provides stability to the anterior horns of the menisci.^[28]^
(3) In some cases, it is necessary to perform femoral notchplasty if the ACL is impinged, as it could block full knee extension.^[29]^

Additionally, deepening the fracture bed and removing the sclerotic bone with an arthroscopic burr are necessary to promote fracture healing.

There are some limitations in this study that must be mentioned. First, the small sample size of this study may have caused a potential bias. Second, a blinded method was not used in this trial, and the stability and function measurements were evaluated by the surgeons themselves. Therefore, larger scale biomechanical studies with a bigger cohort are required in the future to further validate our findings.

In conclusion, arthroscopy-assisted transosseous fixation with the Versalok suture anchor is a minimally invasive and easily accessible procedure for avulsion fracture of the ACL tibial insertion in adults and can result in satisfactory functional recovery. In addition, preoperative MRI is necessary to avoid inadequate preoperative preparation.

## Author contributions

**Investigation:** Yuanshi She.

**Project administration:** Yuanshi She, Youjia Xu.

**Resources:** Guangxiang Chen, Youjia Xu.

**Writing – original draft:** Yuanshi She.

**Writing – review & editing:** Yuanshi She, Dongsheng Guo.
